# The Pandemic and Beyond: Innovation in Cardiovascular Training to Improve Quality of Education and Trainees’ Well-being

**DOI:** 10.14797/mdcvj.1107

**Published:** 2022-06-03

**Authors:** Hyeon-Ju R. Ali, Stephen H. Little, Nadeen N. Faza

**Affiliations:** 1 Department of Cardiology, Houston Methodist Hospital, Houston, Texas, US

**Keywords:** coronavirus-19, cardiovascular training, well-being, career development

## Abstract

During the first 2 years of the coronavirus-19 pandemic, many changes and innovations occurred to overcome the challenges associated with the pandemic and improve cardiovascular training. This review highlights the literature on the pandemic response regarding cardiovascular fellowship education and identifies areas of need to ensure future opportunities for fellows to achieve competency and career advancement. Specifically, we describe the recent changes to the four cornerstones of cardiovascular training: core content education, procedural training, career development, and the well-being of trainees.

## Introduction

The coronavirus-19 (COVID-19) pandemic posed new challenges to medical education and training programs across the spectrum of specialties.^1^ While the medical education system was based on in-person learning, particularly hands-on procedural training as well as personal mentorship both within and across institutions, the social distancing precautions necessitated by the pandemic rendered these familiar methods impractical. Cardiology training programs were one of the specialties that was significantly impacted due to reduction in inpatient volume, elective procedures, and outpatient clinical visits.^2^ In the 2 years since the declaration of the pandemic, there has been remarkable innovation and resilience in response to the challenge of maintaining quality in training and well-being of trainees. In this review, we summarize the existing literature to inform current practices and future responses in training programs.

## Cornerstones of cardiovascular training

The initial impact of the COVID-19 pandemic on cardiovascular training programs was largely concentrated on redeployment necessitated by high volumes of COVID-19–related admissions, which led to a shortage of personal protective equipment (PPE) and strain on the mental and physical health of trainees.^3,4^ With the rapid development of vaccines against COVID-19 and replenished supplies of PPE, the conversation has increasingly shifted toward the question of fulfillment of core competencies and career advancement. Several models of fundamental principles of cardiovascular training have been proposed to assess the impact of the pandemic on the quality of education. Drs. DeFillipis, Schmidt, and Reza were some of the first to outline key components of training programs as well as proposed adaptations to specific challenges posed by the pandemic.^5^ These components included experiential learning, procedural experience, telemedicine, virtual education, leadership, and trainee well-being and safety. Dr. Weissman et al. also noted the challenges in maintaining research mentorship and in identifying potential solutions at the level of regulatory organizations (eg, redefining competency based on individual assessments rather than volumes, such as the American College of Cardiology Core Cardiology Training Symposium, or COCATS, requirements).^6^ Similar “transformational domains” were identified by Chong et al. regarding cardiovascular training in Canada.^7^

**Figure 1:**
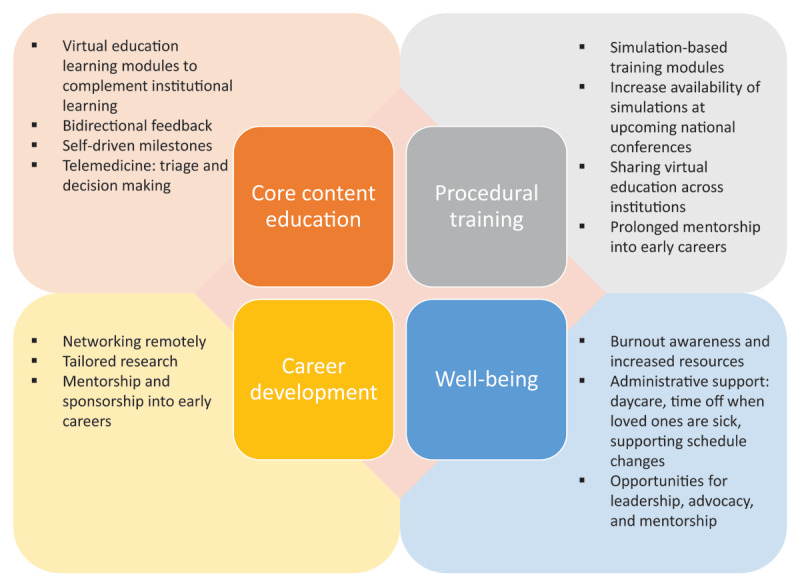
Cornerstones of cardiovascular training and innovative solutions to challenges.

Based on these prior publications, we propose a model for four cornerstones of cardiovascular training (**Figure 1**). As we enter our third year of the pandemic, there are a few considerations to keep in mind. Compared to past years, more incoming fellows may have had less exposure to cardiology electives and fewer opportunities to engage in cardiology-related research.^8^ In addition, some incoming fellows may have had disruptions in their education due to redeployment or potentially less procedural experience.^1^ While virtual education has increased the availability of resources for trainees, they may have missed out on the benefits of in-person education—the engagement, networking, and opportunity for their own presentations at national conferences.^9^ “Zoom fatigue” may be an ongoing challenge for trainees who have trained in this no-contact virtual world for 2 years.^5^ On the other hand, whether transitioning from residency to fellowship or from fellowship to subspecialty fellowship/early career, trainees have had 2 years of experience, or lessons learned, that can inform present and future responses to the pandemic. In the following sections, the four domains of cardiovascular training—core content education, procedural training, career development, and well-being—are discussed in detail.

### Core Content Education

The pandemic highlighted the importance of a clinical skill that was previously underrecognized: competency in telemedicine. According to a national survey of cardiology fellows, 66% were providing telemedicine care and 74% felt that additional training in virtual clinical encounters would be beneficial.^4^ While telemedicine minimizes potential exposures to the virus among those who are unknowingly infected, it has also posed new challenges to our trainees in maintaining quality care. We have learned to work through technological difficulties and account for the lack of in-person physical exams; however, there must be renewed emphasis in taking a proper, detailed history. In addition, the “look test” over the video telemedicine—for example, to determine whether a patient is in distress or in extreme volume overload—constitutes a key physical exam finding. Gathering available data such as heart rates, blood pressures, and weights and utilizing remote monitoring such as event monitors or CardioMEMs is an important aspect of virtual patient care.^10^ The final medical decision making regarding next steps is a complex interplay of the patient’s clinical status and indicated further workup and management, the patient’s personal risk of contracting the virus, and management of limited resources. Even in non-pandemic times, triaging patients based on information available over the phone is an invaluable skill.^10^ Three-way call capabilities to include the fellow, attending physician, and patient as well as allowing adequate time for feedback on tele-visits is essential to ensure patient safety and fellows’ education.^5^

One of the fundamental changes that has occurred during the pandemic is the transition from in-person to virtual education, which has been a shared experience across nearly all programs.^4^ When considering the traditional core content education, the importance of self-assessment to shape curriculum has become even more important during the pandemic. In fact, the recent changes to core programmatic requirements by the American College of Graduate and Medical Education stipulates that trainees engage in self-assessments twice a year with regard to achieving milestones in the six core competencies.^11^ By adding these opportunities for reflection, trainees and program directors can engage in targeted discussions regarding areas in which trainees may require additional training and create individualized learning plans. Furthermore, this exercise allows programs that are particularly impacted by the pandemic to focus on competency-based rather than volume-based learning. These changes promote not only self-actualization but also provide opportunities for program leadership to identify areas that require further investment from the program.

Regarding the transition to virtual education, the responses from fellows have been largely positive; 73% of fellows reported enjoying the virtual learning and hoped to continue having access to online resources even after the pandemic.^4^ In fact, in one survey of pediatric cardiology fellows, almost all of them (93.7%) felt that online learning compared to in-person lectures was as convenient and improved fellows’ attendance (93.0%).^12^ The majority of these respondents also felt that virtual education was equally or more effective in educational value compared to in-person learning.^12^ A review by Senapati et al. in the July 2020 issue of the *Methodist DeBakey Cardiovascular Journal* reported on the importance of social media and virtual learning in education and career development for fellows and early career cardiologists.^13^ National societies, such as the American College of Cardiology and American Heart Association, have made virtual resources, including recorded lectures, available for cardiology fellows to access for free. These have improved access to core content during a time educational conferences at their home institutions were delayed or cancelled.

While there have been many improvements in the delivery of core content education, several challenges have been identified during the first 2 years of the pandemic. Although the increased availability of online resources has improved access, it has also led to a significant increase in screen time,^7,14^ which has been associated with burnout, depression, and anxiety. Also, more in-person communication has been replaced by e-mails, which has increased administrative burden for both fellows and faculty.^15^ While online access has made it more convenient to attend meetings and conferences from anywhere,^12^ the majority of fellows have reported more likelihood of distraction and lower retention of presented material.^16^ Lastly, online platforms can have several inherent limitations, such as reliance on variable quality of Internet connection, interference from background noise, or difficulty hearing from multiple participants at the same time.

There have been several proposed solutions to combat these challenges. To reduce the administrative burdens and stress of increased screen time, some programs have centralized their online presence to one platform, such as Microsoft Teams, which permits sharing of information, scheduling meetings, and holding conferences.^15^ Such centralization, however, requires up-front investment to educate and orient the staff to take advantage of its features across the system. To improve engagement and participation, new features have been put to the test, such as “live polls” during conferences to allow the audience to “control the mouse,” and in-person smaller group gatherings during times of low incidence of COVID.^15^ Other programs have come together locally on virtual platforms to share cases and educational opportunities. The New England consortium of interventional cardiology fellows is one such example; it has had great success in augmenting its training particularly during times of high incidence of COVID and low volumes of procedures.^17^

In one study done during the pandemic, virtual platforms were directly compared to historical in-person classrooms among medical students in clinical radiology rotations.^18^ Students reported less satisfaction with virtual education compared to in-person classrooms as it pertained to didactic quality, engagement of lecturers, and knowledge gained.^18^ However, exam grades were significantly better in the virtual education group compared to in-person group at the end of the rotation.^18^ Of note, this intervention involved a structured format, including prelecture videos that students were expected to watch to prepare for classes and thereby actively participate during the lecture in reading radiographic images themselves. Such methods, titled “heutagogic teaching model of blended learning,” could supplement interpretation of echocardiography and catheter-based angiograms for fellows during virtual training times.^18^ Future studies can investigate how to further optimize virtual education such as the mode of delivery (eg, live versus recorded lectures), type of content best delivered through online education (eg, interpretation of studies versus procedural training), and supplemental resources (eg, pre- and post-tests).

### Procedural Training

With significantly fewer patients presenting with acute cardiac complaints as well as deferral of elective procedures,^19^ the procedural learning opportunities in cardiac catheterization labs have decreased significantly. Between January and March of 2020, 95% of interventional cardiology fellows reported foreseeing a moderate to severe impact on their training that year.^20^ By April 2020, 50% of the fellows reported more than a 75% decline in procedural volumes during the same time period.^21^ The pandemic had a similar impact on transthoracic and transesophageal echocardiographic image acquisition training. In a survey by Rao et al., 69% of fellows reported acquiring fewer transthoracic echocardiograms and 88% reported a decrease in echocardiography lab staffing.^4^ Given the necessary social distancing precautions, stress tests had been suspended at various times,^22^ leading to fewer opportunities for fellows to oversee stress tests and respond to stress lab emergencies. Currently, there are no studies describing the impact of COVID on the class of fellows beginning their training in July 2021. Future investigations are needed to better understand how the impact of the COVID-19 pandemic may have evolved over time and, in particular, differed across geographic regions.

Despite the training limitations imposed by the pandemic, cardiology fellows must still follow the training guidelines established in the 2015 COCATS requirements, which are the most current.^23^ **Table 1** shows the suggested minimum number of procedures and examinations required to be considered proficient at each level of training in catheterization, echocardiography, and nuclear medicine. Because fellows have faced significant challenges meeting such volume-based requirements, the National Board of Echocardiography extended the amount of time available to obtain requisite procedural experience by 1 year for those unable to complete the required volume due to the pandemic.^24^ On the other hand, some have called on these governing boards to reduce stringent procedural volume requirements.^25,26^ In fact, it is important to note that the 2015 COCATS authors emphasized a shift toward *competency*-based training from a minimum volume-based requirement.^27^ In addition to learning the technical skills of a particular procedure, fellows are expected to demonstrate proficiency in medical knowledge, patient care, practice learning and improvement, interpersonal and communication skills, and professionalism.^23^ These are areas of competency that may be best addressed on an individual level, with self-assessment and bidirectional feedback between the fellow and program directors.

**Table 1:** American College of Cardiology Core Cardiology Training Symposium requirements for proficiency levels in catheterization, echocardiography, and nuclear medicine. TTE: transthoracic echocardiogram, TEE: transesophageal echocardiogram


FIELD	LEVEL 1	LEVEL 2	LEVEL 3

Catheterization	4 months 100 diagnostic procedures 50 coronary angiographies 25 valvular, myocardial, pericardial, congenital procedures	6 months 300 diagnostic procedures Peripheral vascular: 100 diagnostic procedures	Additional year No specific minimum requirement noted

Echocardiography	3 months 75 TTE exams performed 150 TTE exams interpreted	6 months 150 TTE exams performed 300 TTE exams interpreted	150 TTE exams performed 750 TTE exams interpreted 150 TEE exams performed and interpreted 3D TTE or TEE: 50 for valve disease, 50 for ventricular volumes, function, ejection fraction 100 contrast TTEs 50 strain and strain rate quantifications 200 stress TTEs including 25 for noncoronary indications

Nuclear cardiology	2 months 100 examinations	4 months 300 exams interpreted	Additional year No specific minimum requirement noted


At the end of the day, technical skills and understanding of complications are best learned through in-person training opportunities. Simulation-based training (SBT) to increase hands-on experiences during peak incidence has gained interest during the pandemic. Even prior to pandemic times, SBT for performing coronary angiograms was noted to provide an advantage over standard training without simulation.^28,29^ In one randomized controlled trial with 27 cardiology fellows, SBT for performing a selective coronary angiogram led to significant improvement in technical and global performance as determined by an attending cardiologist who was blinded to the intervention.^28^ Psychomotor skills and hand-eye coordination involved in obtaining intravascular access, mounting the catheter over the wire, advancement of the catheter, and engagement of coronary arteries were all found to improve after SBT-based training.^28^ A similar virtual training environment was tested for training echocardiography image acquisition at the University of Texas Southwestern cardiology program.^30^ The simulation-based scanning facilitated remote bidirectional feedback between advanced sonographers and trainees and improved performance based on a checklist of basic images and targeted disease processes, such as pericardial effusion and aortic stenosis.^30^

It is unclear whether SBT and web-based conferences can adequately compensate for the loss of in-person learning opportunities during the pandemic. While it seems unfair to lengthen fellowships, many graduating fellows do report concerns regarding their competencies in procedures due to limited training opportunities during the pandemic. Interestingly, in a 2020 survey of 135 interventional cardiology fellows and 152 program directors, 49% of the fellows felt their procedural competence was impaired while 97% of program directors felt their fellows would be procedurally competent.^17^ This discordance highlights the importance of a feedback loop to not only identify areas for improvement but also help graduating fellows build confidence in their skillset. SBT may help to increase “face-to-face” time with the instructors, even if remotely, and allow fellows to reach their own self-driven milestones by graduation. Unfortunately, according to a survey done in 2020, only 20% of cardiology fellows have access to simulation centers.^4^ With some national conferences returning to in-person meetings, there may be opportunities for the industries to provide additional simulation center opportunities for fellows who can attend in person. Moreover, during the next few years, it may be particularly important for graduating fellows to have prolonged mentorship into their early careers and continue on their self-assessment learning plans with the help of chosen mentors.^5^

### Career Development

This year, the cardiology fellows who are graduating in 2023 are preparing their subspecialty fellowship or job applications. From their very first year of training, which began in July 2020 prior to vaccine availability, this class of fellows experienced impaired in-person learning opportunities due to the pandemic. Fellows reported being concerned about their early career prospects as early as 2019, when the pandemic first appeared.^4^ With the ensuing reduction in procedural volumes, there were hiring freezes in some areas, and interventional cardiologists reported limited job opportunities.^17^ There has been a significant amount of literature generated regarding the residency match process, particularly in competitive specialties such as dermatology, radiology, and surgery. Of note, while there are concerns about limited networking due to restrictions in away rotations and in-person national conferences, the impact on the residency match has been mixed. One survey of 2020/2021 applicants to plastic surgery found that applicants were more likely to match to programs where they had prior connections (including home institutions),^31^ while another survey of otolaryngology applicants and program directors found that there was no significant geographic clustering.^32^ The real impact of the pandemic on the cardiology subspecialty fellowship match or the hiring process remains unknown and is an important area for future research.^17^

In many cases, social distancing requirements prevented fellows from pursuing their usual networking opportunities and, in some cases, from completing their clinical research. The majority of cardiology fellows (62%) reported cancelling attendance at national conferences, with 24% stating that they even cancelled their own presentations.^4^ To address such challenges, our national societies have increased their efforts to promote networking remotely and to create options to attend national conferences virtually. The ability to present virtually may increase opportunities for fellows to participate by reducing the required travel expenses and the time away from clinical responsibilities. During their 2021 national conference, the ACC held a “virtual career fair” in which fellows could connect with mentors and other like-minded fellows based on their interests. An increase in other virtual gatherings occurred across local and state chapters in 2021, such as a women-in-cardiology meet and greet event held by the Texas ACC chapter. The American Heart Association maintains an updated database of available jobs as well as access to over $100 million in research funding. While the pandemic may have slowed down clinical trials that require patient enrollment, the lower clinical volumes can also afford fellows the opportunity to engage in other types of research, such as literature reviews and retrospective studies^9^ as well as COVID-19–related research.^33^ Even as clinical volume returns and distancing requirements subside enough to allow in-person national meetings, these resources may continue to benefit fellows to further their career interests. Future investigations may help to identify needs and resources for cardiology fellows and trainees with regard to additional skills needed to network in the virtual space, whether via email or videoconferencing.

### Well-being

The impact of COVID-19 on trainees’ mental health was recognized early in the pandemic.^34^ Particularly with limited availability of PPE, 81% of fellows reported concerns about personally contracting COVID-19 and 87% were concerned about transmitting the virus to their loved ones.^4^ Burnout became more prevalent and was significantly associated with loss of learning opportunities, unpredictable schedules, and lack of procedural volumes to ensure competency prior to graduation.^1,35^ These challenges were met with a renewed focus on support for the trainees—whether it was time off from work to care for sick loved ones, schedule changes to accommodate needs, or increased child support when community resources were closed.^2,6,36^

The availability of virtual options for conferences, patient clinic visits, and coordination of care has the potential to help trainees become more efficient and improve life-work balance as long as it does not increase the burden of unnecessary documentation and meetings. The pandemic saw increased social media presence among fellows and early career cardiologists, which provided a sense of community and assuaged fears as we tackled difficult challenges such as optimal management of ST-elevation myocardial infarction during COVID-19 surges.^36^ Importantly, mental health conditions such as depression and anxiety among healthcare workers became more recognized during the pandemic, leading to creation of more accessible mental health resources.^2^ Kotta et al. summarized resources that are available to support physician well-being, such as Headspace, Breathe2Relax, and Insight Timer.^37^ In this regard, program leadership can serve an important role as advocates for trainees. By creating layers of backup, fellows were able to take the time needed for their loved ones or themselves as they cope with new life challenges from the pandemic.^36^ Interprogram leadership, such as the Committee of Interns and Residents, advocated for adequate PPE and hazard pay. In our own hospital, when the cardiac intensive care unit (ICU) had to be repurposed for COVID-19 patient care, our fellows were reassigned to cardiac floors and cardiovascular ICUs to continue their core cardiovascular training while limiting their exposures.

## Conclusion

The COVID-19 pandemic has necessitated several changes to cardiovascular training paradigms to compensate for the large reduction in clinical volumes and training opportunities. However, the pandemic has also encouraged rapid exchange of ideas in best practices for maintaining educational excellence in our cardiovascular training programs. While we continue to face high levels of uncertainty with regard to COVID-19, our fellows and program leadership across the country continue to rise to the challenge. Ultimately, online resources, a renewed focus on individualized learning plans, and programmatic changes to prevent burnout are positive changes that will continue to benefit us beyond the pandemic times. Ongoing innovation, particularly with regard to procedural learning, and increasing access to simulation-based training in catheterization and echocardiography labs are important areas for future research.

## Key points

- Cardiovascular training programs have developed innovative solutions to the challenges posed by the reduction in clinical volumes and necessary contact precautions during the COVID-19 pandemic.
- Educational content available via website has democratized and improved access to learning resources for all fellows; however, effective procedural training still remain a challenge.
- Ongoing efforts are needed to maintain and improve upon burnout prevention strategies and increase collaboration/networking opportunities within and across different cardiovascular programs.
- Future studies are needed to better understand how some of these mitigation strategies have improved the learning and career development opportunities for fellows-in-training during the last year of COVID-19 pandemic.
